# Audit-identified avoidable factors in maternal and perinatal deaths in low resource settings: a systematic review

**DOI:** 10.1186/1471-2393-14-280

**Published:** 2014-08-16

**Authors:** Hasan S Merali, Stuart Lipsitz, Nathanael Hevelone, Atul A Gawande, Angela Lashoher, Priya Agrawal, Jonathan Spector

**Affiliations:** The Hospital for Sick Children, 555 University Avenue, Toronto, ON M5G 1X8 Canada; Center for Surgery and Public Health, Brigham and Women’s Hospital, Boston, MA USA; Ariadne Labs: A Joint Center at Brigham and Women’s Hospital and Harvard School of Public Health, Boston, MA USA; World Health Organization, Geneva, Switzerland; Harvard School of Public Health, Boston, MA USA; MassGeneral Hospital for Children, Boston, MA USA

**Keywords:** Maternal, Fetal, Neonatal, Perinatal, Avoidable, Factors, Death, Mortality

## Abstract

**Background:**

Audits provide a rational framework for quality improvement by systematically assessing clinical practices against accepted standards with the aim to develop recommendations and interventions that target modifiable deficiencies in care. Most childbirth-associated mortality audits in developing countries are focused on a single facility and, up to now, the avoidable factors in maternal and perinatal deaths cataloged in these reports have not been pooled and analyzed. We sought to identity the most frequent avoidable factors in childbirth-related deaths globally through a systematic review of all published mortality audits in low and lower-middle income countries.

**Methods:**

We performed a systematic review of published literature from 1965 to November 2011 in Pubmed, Embase, CINAHL, POPLINE, LILACS and African Index Medicus. Inclusion criteria were audits from low and lower-middle income countries that identified at least one avoidable factor in maternal or perinatal mortality. Each study included in the analysis was assigned a quality score using a previously published instrument. A meta-analysis was performed for each avoidable factor taking into account the sample sizes and quality score from each individual audit. The study was conducted and reported according to PRISMA guidelines for systematic reviews.

**Results:**

Thirty-nine studies comprising 44 datasets and a total of 6,205 audited deaths met inclusion criteria. The analysis yielded 42 different avoidable factors, which fell into four categories: health worker-oriented factors, patient-oriented factors, transport/referral factors, and administrative/supply factors. The top three factors by attributable deaths were substandard care by a health worker, patient delay, and deficiencies in blood transfusion capacity (accounting for 688, 665, and 634 deaths attributable, respectively). Health worker-oriented factors accounted for two-thirds of the avoidable factors identified.

**Conclusions:**

Audits provide insight into where systematic deficiencies in clinical care occur and can therefore provide crucial direction for the targeting of interventions to mitigate or eliminate health system failures. Given that the main causes of maternal and perinatal deaths are generally consistent across low resource settings, the specific avoidable factors identified in this review can help to inform the rational design of health systems with the aim of achieving continued progress towards Millennium Development Goals Four and Five.

**Electronic supplementary material:**

The online version of this article (doi:10.1186/1471-2393-14-280) contains supplementary material, which is available to authorized users.

## Background

Devising strategies that measurably improve maternal and newborn care in low resource settings is an urgent global priority [1, 2]. Nearly 300,000 maternal deaths [3], 3 million newborn deaths [4], and 1 million intrapartum-related stillbirths [5], take place each year in grossly disproportionate geographic patterns. Given that there are many countries with very low childbirth-related mortality rates, it is clear that high childbirth-related mortality burdens are not inevitable. Rational bolstering of health systems saves lives, even in lower income settings where resources are limited.

The major complications that result in maternal, newborn, and fetal deaths are well described. For mothers, these are traditionally categorized as excessive hemorrhage, infection, hypertensive disorders, and obstructed labor [6]. For babies, these are intrapartum-related events (previously called birth asphyxia), infection, and complications of prematurity [7]. Avoidable stillbirths are largely attributed to inadequate intrapartum care [5]. These categories provide an important orientation to the general causes of childbirth-related deaths and as such are fundamental to establishing a basis for strengthening health systems. However, a limitation of these somewhat broad categorizations is insight into where exactly deficiencies in clinical care are occurring, information that is critical to the design and implementation of effective health system improvements. For example, a maternal death from hemorrhage can result from absent prophylactic oxytocin, undetected bleeding, and/or inaccessible blood transfusion capability—three different types of system failures that necessitate different intervention approaches to prevent failure recurrence. Targeted health system strengthening relies on a systematic analysis of the events that lead to deaths in order to determine if avoidable breakdowns in medical care are present. If such deficiencies exist, fully characterizing them and pinpointing precisely where in the clinical care continuum they occur provides clinicians, policymakers, and other stakeholders with information needed to effectively address them.

Audits are tools that provide a logical framework for quality improvement by systematically assessing clinical practices against accepted standards [8]. Mortality audits have demonstrated success in helping to reduce childbirth-related deaths in lower income countries [9–11]. Since the main causes of maternal and perinatal deaths are consistent across lower income countries, it stands to reason that there are also similarities in the avoidable factors associated with those deaths.

Most maternal and perinatal death audits have been restricted to a single facility or region with no widely utilized centralized mechanism for aggregating data from across countries [12–14]. Up to now, the avoidable factors in deaths cataloged in these reports have not been comprehensively pooled and analyzed. We sought to identify the most frequently reported factors in avoidable childbirth-related deaths globally through a systematic review of all published reports of mortality audits in low and lower-middle income countries. Our main objectives were to identify those factors that repeatedly account for high proportions of avoidable maternal and perinatal deaths in individual audits as well as to identify the avoidable factors that contribute to the most deaths overall.

## Methods

### Eligibility

Studies from low and lower-middle income countries were considered, using World Bank criteria for stratification of country by gross national income (GNI) per capita [15]. Studies were eligible if they performed an audit of maternal or perinatal deaths using medical records, meetings of health workers, and/or interviews of health workers or patient families. Studies had to explicitly identify at least one avoidable factor in a maternal or perinatal death, and studies had to utilize definitions for maternal and perinatal deaths that were reasonably similar to those used by WHO. Maternal deaths are defined by WHO as “the death of a woman while pregnant or within 42 days of termination of pregnancy, irrespective of the duration and site of the pregnancy, from any cause related to or aggravated by the pregnancy or its management but not from accidental or incidental causes” [16]. The perinatal period, as defined by the WHO, “commences at 22 completed weeks (154 days) of gestation and ends seven completed days after birth” [17]. Perinatal mortality refers to stillbirths and newborn deaths in the first week of life [17]. For the purpose of this analysis, a factor in an “avoidable death” was defined as one that was assessed to be directly related to the death; in other words, if the factor had been avoided than the death would probably not have occurred [18]. Audits that did not report specific avoidable causes in maternal and/or perinatal deaths were excluded. Two investigators (HSM and JS) determined the eligibility of the articles independently and any discrepancy was resolved by a discussion between these two investigators.

### Search strategy

We performed a systematic search of published literature from 1965 to November 2011 in Pubmed, Embase, CINAHL, POPLINE, LILACS and African Index Medicus. The search strategy included various combinations of exploded and focused MeSH headings and keyword searching using the terms Perinatal, Maternal, Mothers, Neonatal, Newborn, Infant, Mortality, Audit, Clinical Audit, Death, Fatal Outcome, Avoidable, Preventable, and Developing Countries. A previously described “snowball” search strategy was also performed in which papers were identified through bibliographies of key studies [19]. We also attempted to identify additional relevant studies, in particular non-published datasets, through queries to experts and international organizations concerned with maternal and perinatal health. Articles in all languages were considered and translated when necessary.

### Quality scoring

Each article that met inclusion criteria was assigned a quality score in order to impose a weighing scheme according to the quality of the study. The score, developed to analyze the quality of obstetrical care in low and middle income countries [20], utilizes a numerical scale ranging from zero (lowest quality) to nine (highest quality). Calculation of the quality score was conducted by assessing the following criteria: selection of audit cases (maximum 3 points), quality control during the audit process (maximum 4 points), and reliability of the audit (maximum 2 points) (See Nine-point clinical audit quality criteria). Two investigators (HSM and JS) independently scored each audit by assigning one point for each criterion met by the article. If there was a discrepancy between the scores of the two investigators, the two scores were averaged to arrive at a final score.

**Nine-point clinical audit quality criteria adapted from Pirkle*et al.*** [20].

- Selection of cases
  - Description of study population with clear case definition
  - Description of sampling strategy
  - Consideration of missing cases
- Data quality control
  - Criterion-based clinical audit pilot or pre-tested
  - Description of staff profile
  - Training of staff
  - Data entry validity checks
- Reliability
  - Standardized data collection form
  - Inter-observer/inter-site variability assessed

### Statistical analysis and reporting

A meta-analysis was performed for each avoidable factor identified. First, we calculated the percent of studies where the factor was determined to be a cause of a maternal or perinatal death. Next, for those studies where the factor was determined to be a cause of death, a “pooled” or combined estimate of the percentage of deaths in those studies due to the factor was calculated. The pooled estimate was calculated using a weighted average of the estimates from the individual studies via accepted meta-analysis techniques [21, 22], in which the study is treated as a random effect. We estimated the weighted averages, confidence intervals for the weighted averages, and the variance of the random study effect using an iteratively reweighted least squares approach [21]. The weights used in the iteratively reweighted least squares approach takes into account the sample size from each individual study, the variance of the random study effect, and the quality score of each study. This approach is semi-parametric in that is gives unbiased estimates regardless of the type of outcome variable (continuous, categorical or percentages). Finally, we determined the total number of attributable deaths for each avoidable factor. The study was conducted and reported according to PRISMA guidelines [23], (See Additional file 1). All analyses were performed using SAS/STAT 9.3 (SAS Institute, Cary, NC, 2011).

## Results

### Search results

**Figure 1:**
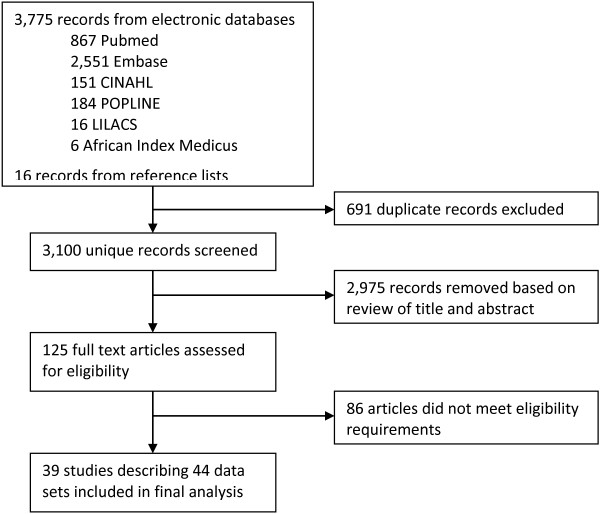
**Literature search flowchart.**

The search returned 3,775 results and an additional 16 papers were added from reference lists. There were 691 duplicate entries and 2,975 were subsequently removed after abstract screening. The full texts of the 125 remaining articles were reviewed and 39 met inclusion criteria (Figure 1). These 39 studies included a total of 44 datasets since five studies included multiple datasets; one study included datasets from two different time periods [24], one study included datasets from two different countries [25], and two studies included datasets from both maternal and perinatal populations [24, 26]. Two other studies analyzed deaths from different time periods [27, 28] but the time periods were contiguous in these studies and so they were considered as one dataset each. We were unable to identify unpublished audits of maternal and newborn deaths that met inclusion criteria. Therefore, only published data were used. We summarized the datasets and categorized them by type of measure examined [24–62] (Table 1).

**Table 1:** **Summary of study datasets (n = 44)**

Authors	Country	Year	Population	Mortality rate/ratio in sample	No. of audits	% avoidable mortality	Methods	Quality score
Ozumba BC, Nwogu-Ikojo EE [29]	Nigeria	2003-2005	Maternal	2,397/100,000	47	70	Retrospective case record review at a university-associated tertiary care hospital	4
Suprakito G, Wirth ME, Achadi E [30]	Indonesia	1998-1999	Maternal	-	130	-	Prospective case record review, staff interviews, and verbal autopsy in 3 districts comprising 5 hospitals and 55 community health centers	5.5
Jacques S, Edgard-Marius O, Bruno D [31]	Benin	2003	Maternal	1,735/100,000	231	55-72	Retrospective case record review at 4 referral hospitals	5
Vangeenderhuysen C, Banos JP, Mahaman T [32]	Niger	1993-1994	Maternal	1,547/100,000	25	84	Prospective case record review, staff interviews, and family interviews in a group of urban hospitals	4
El Amin S, Langhoff-Roos J, Bodker B, *et al*. [33]	Sudan	2000	Perinatal	82/1,000	43	58-82	Prospective external audit by multidisciplinary team through case presentations and grading forms at a maternity hospital	7.5
Mbaruku G, van Roosmalen J, Kimondo I, *et al*. [34]	Tanzania	2002-2004	Perinatal	38/1,000	200	-	Retrospective audit by case record review and family interviews at a regional hospital	5.5
Bouvier-Colle MH, Ouedraogo C, Dumont A [35]	West Africa	1994-1996	Maternal	311/100,000	55	69	Prospective survey using questionnaires and verbal autopsy in 6 West African countries: Ivory Coast, Mali, Niger, Mauritania, Burkina Faso, and Senegal. Deaths occurred in hospitals, in health centers, and at home	6
Chigbu CO, Okezie OA, Odugu BU [36]	Nigeria	1999-2007	Perinatal	89/1,000	316	-	Retrospective case record review, physician interview, and midwife interview at a university teaching hospital	3
van Roosmalen J [37]	Tanzania	1971-1976	Perinatal	48/1,000	137	25	Retrospective case record review at a district hospital	2
De Muylder X [38]	Zimbabwe	1984-1986	Perinatal	31/1,000	319	76	Prospective medical record review, family interviews, laboratory evaluation, and necropsy at 6 peripheral birth centers and a referral district hospital	3
Cham M, Vangen S, Sundby J [39]	Gambia	2002	Maternal	279/100,000	42	“Majority”	Prospective medical record review, health worker interviews, and verbal autopsy in one rural district involving 17 birth facilities and one hospital	6
Dumont A, Tourigny C, Fournier P [40]	Senegal	2004-2005	Maternal	-	69	48	Prospective medical record review, health worker interviews, and family interviews at 5 referral hospitals	7
Frost O [26]	Ethiopia	1980	Maternal	780/100,000	30	-	Prospective medical record review and health worker interviews at a national referral hospital	4
Frost O [26]	Ethiopia	1980	Perinatal	9/1,000	291	8	Prospective medical record review and health worker interviews at a national referral hospital	4
Price TG [41]	Tanzania	1983	Maternal	250/100,000	115	-	Prospective case record review and questionnaires in a region comprising of hospitals, health centers, and dispensaries	5
Adetoro OO [42]	Nigeria	1972-1983	Maternal	450/100,000	624	-	Retrospective medical record review at a national referral center	3
Bullough CH [43]	Malawi	1977	Maternal	263/100,000	109	88	Prospective questionnaires completed by physicians and midwives at 1 central hospital, 8 district hospitals, 6 mission hospitals, and 92 rural birth facilities	4
Johnstone FD, Ochiel SO [44]	Kenya	1976-1977	Perinatal	97/1,000	393	-	Retrospective case record review at a national referral hospital	3
Hinderaker SG, Olsen BE, Bergsjo PB, *et al*. [45]	Tanzania	1996-1996	Perinatal	27/1,000	136	51-65	Prospective interviews of antenatal care attendees combined with retrospective household surveys using verbal autopsy and medical records in 7 rural communities. Deaths occurred at home and in rural health facilities.	7
D’Ambruoso L, Byass P, Qomariyah SN, *et al.* [25]	Indonesia	2002-2006	Maternal	-	104	-	Retrospective review of verbal autopsies in a community with a district hospital and 19 health centers	6
D’Ambruoso L, Byass P, Qomariyah SN, *et al.* [25]	Burkina Faso	2002-2006	Maternal	-	70	-	Retrospective review of verbal autopsies in a community with 5 hospitals and 66 health centers	6
Hailu S, Enqueselassie F, Berhane Y [46]	Ethiopia	2005-2006	Maternal	-	34	35	Retrospective case record review and health worker interviews in 5 public hospitals	5.5
Issah K, Nang-Beifubah A, Opoku CF [47]	Ghana	2009	Maternal	-	47	49	Prospective questionnaires completed by health workers in a community with 6 hospitals and 73 health centers	5
Jafarey S, Rizvi T, Koblinsky M, *et al.* [48]	Pakistan	2005-2007	Maternal	-	128	-	Retrospective and prospective verbal autopsy in two districts at the community and hospital levels	8
Kongnyuy E, Mlava G, van den Broek N [49]	Malawi	2007	Maternal	-	43	-	Prospective register review, referral note review, case record review, and family interviews in 9 hospitals	5
Waiswa P, Kallander K, Peterson S, *et al*. [50]	Uganda	2005-2008	Perinatal	-	64	-	Retrospective case record review from volunteer collected data, and a standard verbal autopsy questionnaire. Study included home and hospital deaths.	7
Sorensen BL, Elsass P, Nielsen BB, *et al*. [51]	Tanzania	2006-2008	Maternal	549/100,000	62	-	Retrospective case record review, staff observations, and staff interviews at a regional hospital	4.5
Lori JR, Starke AE [52]	Liberia	2008	Maternal	-	28	-	Prospective case record review using a standard audit tool, combined with interviews conducted by trained nurses at the community and hospital level	6
Granja AC, Machungo F, Bergstrom S [53]	Mozambique	1989-1990	Maternal	340/100,000	106	40	Retrospective medical record review at an urban referral hospital	3
Granja AC, Machungo F, Gomes, A, *et al*. [54]	Mozambique	1989-1993	Maternal	320/100,000	239	75	Retrospective medical record review at an urban referral hospital	2.5
Kidanto HL, Mogren I, van Roosmalen J, *et al*. [55]	Tanzania	2007	Perinatal	92/1,000	133	52-75	Retrospective multidisciplinary audit panel of internal and external reviewers using a structured assessment protocol and grading form at a national hospital	7
Olsen BE, Hinderaker SG, Bergsjo P, *et al*. [56]	Tanzania	1995-1996	Maternal	-	45	31	Retrospective and prospective review of hospital records, village leader reported deaths, household surveys and antenatal clinic records at the community and hospital level. Verbal autopsy questionnaires were also used.	5.5
Oladapo OT, Ariba AJ, Odusoga OL [28]	Nigeria	1999-2004	Maternal	2508-2931/100,000*	71	-	Retrospective case record review at tertiary care hospital. Reviewed by committee of 3 consultants and 3 residents.	4
Byaruhanga RN [57]	Uganda	1997-1998	Perinatal	68/1000	235	-	Prospective case record review by a team comprised of a pediatrician, obstetrician and three midwives at a tertiary referral hospital	5
De Muylder X [58]	Zimbabwe	1985-1987	Maternal	137/100,000	70	50	Retrospective case record review by a multidisciplinary committee of deaths occurring at district and rural hospitals	3.5
Bhatt RV [24]	India	1967-1968	Maternal	1,448/100,000	43	10	Prospective case record review combined with staff meetings and interviews of caregivers at a university teaching hospital	6
Bhatt RV [24]	India	1967-1968	Perinatal	115/1,000	342	-	Prospective case record review combined with staff meetings and interviews of caregivers at a university teaching hospital	6
Bhatt RV [24]	India	1983-1984	Maternal	1,152/100,000	36	1.5	Prospective case record review combined with staff meetings and interviews of caregivers at a university teaching hospital	6
Bhatt RV [24]	India	1983-1984	Perinatal	101/1,000	315	-	Prospective case record review combined with staff meetings and interviews of caregivers at a university teaching hospital	6
Mbarku G, Bergstrom S [27]	Tanzania	1984-1991	Maternal	186 – 933/100,000*	132	-	Retrospective and prospective case record review with interventions in 1986 at a regional hospital	3
Steklenberg J, van Roosmalen J [59]	Zambia	1999-2001	Maternal	1,359/100,000	15	-	Prospective case record review and regular maternal mortality review meetings at a district hospital.	4.5
Ouedraogo C, Bouvier-Coller MH [60]	Burkinia Faso, Ivory Coast, Mauritania, Mali, Niger and Senegal	1985-1997	Maternal	Variable by country	55	-	Retrospective case record review combined with “verbal autopsy” using a questionnaires and a multidisciplinary team review of all cases at the hospital level	5
Qazi GR [61]	Pakistan	1992	Maternal	-	40	-	Retrospective case record review at a university teaching hospital	3
Rachid B, Abouchadi S, de Brouwere V, Belghiti A [62]	Morocco	2009	Maternal	-	436	76	Retrospective case record review for healthcare setting deaths, and verbal autopsy for home deaths	2.5

*2 calculations covering two different time periods within a continuous time period.

### Dataset characteristics

Forty-four datasets were included in the study, comprising a total of 6,205 audited deaths. The majority of the datasets, 82%, came from African studies. Two datasets (4.5%) were from Pakistan [48, 61], two (4.5%) from Indonesia [25, 30], and four (9.1%) from India [24]. Of note, the four datasets from India were obtained from the same investigation [24]. The oldest dataset was from 1967 [24] and the most recent datasets were from 2009 [47, 62]. Thirty-one of the 44 datasets (70.5%) examined maternal deaths while the other 13 datasets (29.5%) examined perinatal deaths. Study methods varied and there were an equal number of datasets that employed prospective methods (45.5%) as those that used retrospective methods (45.5%). Four of the datasets [27, 45, 48, 56] (9.1%) used a combined retrospective and prospective methodology. The majority of the datasets, 72.7%, described hospital populations, while the remaining 27.3% described both hospital and community populations. Many of the studies reported the maternal mortality rate and perinatal mortality rate from their audited populations, and these varied between studies. Just under half of the studies (47.7%) estimated the percentage of deaths that were thought to be avoidable in the sample audited, and this ranged from 1.5% to 88%. Quality scores for the audits ranged between 2–8 (See Table 1).

### Avoidable factors

Overall there were 42 avoidable factors in maternal and perinatal deaths identified from the 44 datasets. These 42 factors fell into four general categories: health worker-oriented factors were the most common, accounting for 28 (66.7%) of the 42 factors; the next most common, in descending order of frequency, were patient-oriented factors (14.3%), administrative/supply factors (11.9%), and transport/referral factors (7.1%). All of the factors are summarized by category in Table 2 (within each category, factors are listed in order of relative number of attributable deaths).

**Table 2:** **Summary of the 42 avoidable factors, listed by category and in descending order of attributable deaths**

Health worker-oriented factors	Patient–oriented factors	Administrative/supply factors	Transport/ referral factors
Substandard health worker practice	Patient delay	Poor blood transfusion capacity or inappropriate administration	Unidentified lack or delay in transport
Delay in care on admission to birth facility	Poor antenatal care	Medication shortage	Poor transport between facilities
Delayed operative delivery	Use of herbal medicine	General supply/ equipment shortage	Poor transportation from home to facility
Inadequate intrapartum monitoring of mother/fetus	Cultural inhibitions causing delay in seeking care	Unsanitary environment	
Inadequate initial maternal assessment and management	Financial constraints	Inadequate operating theatre facilities	
Unavailability of health worker for key intervention	No knowledge of danger symptoms		
Poor communication between health workers			
Missed or unskilled breech delivery			
Substandard health worker antenatal care practices			
Inadequate monitoring of mothers in hypovolemic or septic shock			
Poor neonatal resuscitation			
Inadequate management of hypertensive related disorders			
Failure to diagnose/ treat neonatal infection			
Failure to diagnose preterm labor			
Failure to diagnose/ treat syphilis			
Health worker related referral delay			
Inadequate management of 3^rd^ stage of labor			
Poor postpartum maternal monitoring			
Inappropriate indication for operative delivery			
Inadequate partogram usage			
Failure to diagnose/treat maternal/fetal infection			
Anesthesia complications during operative delivery			
Inadequate response to poor labor progress			
Inadequate action taken for fetal distress			
Inadequate assessment of fetal distress			
Inadequate assisted vaginal delivery			
Inappropriate discharge when patient not well			
Health worker industrial strike			

The top 10 avoidable factors, listed in order of attributable deaths, are shown in Table 3. The number of deaths attributable to these factors ranged between 251 – 688 of the total sample of 6,205 audited deaths. Each of the top 10 avoidable factors was reported in a maternal or perinatal death in 10 – 23 (22.7% - 52.3%) of the datasets. The most common factors reported overall were patient and transport delays, each of which was a reported factor in 52.3% of the datasets. Health care worker-oriented factors were the most common category of factors among the top 10 factors, accounting for six out of ten factors. Substandard health worker practice was responsible for the most deaths (688 deaths), and we estimated that this factor contributed to 28.5% of the deaths in datasets where it was identified as an avoidable factor (See Additional file 2).

**Table 3:** **Top 10 audit-identified avoidable factors in maternal and perinatal deaths**

Factor	Category	Datasets in which factor was an identified cause of death; n = 44 (%)	Estimate of the factor’s contribution to deaths in datasets in which it was identified; % (CI)	Total number of attributable deaths due to factor in the entire sample; n = 6205 (%)
Substandard health worker practice	Health worker-oriented factor	18 (40.9)	28.5 (19.5 – 39.7)	688 (11.1)
Patient delay	Patient-oriented factor	23 (52.3)	22.2 (16.0 – 30.0)	665 (10.7)
Poor blood transfusion capacity or inappropriate administration	Administrative/ supply factor	21 (47.7)	24.9 (18.5 – 32.6)	634 (10.2)
Delay in care on admission to birth facility	Health worker-oriented factor	20 (45.5)	26.6 (18.5 – 36.6)	628 (10.1)
Undefined lack of or delay in transport	Transport/ referral factor	23 (52.3)	23.7 (16.2 – 33.4)	546 (8.8)
Delayed operative delivery	Health worker-oriented factor	12 (27.3)	23.1 (11.0 – 42.1)	442 (7.1)
Inadequate intrapartum monitoring of mother/fetus	Health worker-oriented factor	10 (22.7)	24.3 (13.8 – 39.1)	374 (6)
Inadequate initial maternal assessment/ management	Health worker-oriented factor	10 (22.7)	20.0 (10.5 – 34.7)	339 (5.5)
Poor antenatal care	Patient-oriented factor	11 (25.0)	14.3 (7.0 – 27.0)	301 (4.9)
Unavailability of health worker for key intervention	Health worker-oriented factor	10 (22.7)	22.4 (10.6 – 41.2)	251 (4)

## Discussion

To our knowledge, this is the first systematic review of avoidable factors in global maternal and perinatal deaths identified by mortality audits. Numerous avoidable factors were identified and found to be related to the behaviors and practices of both health workers and patients, as well as to administrative, supply, referral and transport problems. Chronologically, factors took place across the continuum of childbirth from the antenatal period, through labor and delivery, and into the postpartum and postnatal periods, though the majority of factors were clustered in the intrapartum period.

The most important avoidable factor by attributable deaths was substandard practice by health workers. While it would have been beneficial for audits to describe more precisely the specific substandard practices, there remains value in knowing that the majority of deaths were thought to have been avoidable if health workers performed better. This has implications for current strategies focused on assuring the presence of skilled health workers at every delivery, including campaigns that incentivize women to deliver in health facilities [63–66]. Our data are consistent with the idea that the presence of health workers at deliveries does not alone ensure the safe care of women and newborns. Adequate health worker training is vital and, presumably, so too are refresher courses and patient safety and quality initiatives that help to ensure that minimum standards of care are reliably delivered by health workers at each and every birth. While the audits specifically refer to substandard health worker practice, future work might include efforts to better understand how health care workers are supported or disempowered by the larger health system.

The second most important avoidable factor by attributable deaths was patient delay. Cultural issues and failure by women and their communities to recognize danger signs proved to be significant factors in maternal and perinatal deaths. Detailed descriptions of the specific reasons for patient delay in the studies included delays due to decision-making control by the ‘head of household,’ childcare concerns for other children in the family, mistrust of the health system, and peer pressure by other members of the community. The data suggest that education in the antenatal period should not be reserved for women alone, but also made available to other members of her family and even the community-at-large, parties which may exert influence over the timing of the woman’s presentation to skilled care. This is supported by a recent study in Kenya that also found strong a strong link between women’s structure of social support and likelihood of institutional delivery [67].

The third most important avoidable factor by attributable deaths related to blood transfusion capacity. Specific reasons cited included lack of accessibility to a blood bank, lack of materials for blood collection, blood safety concerns, recruitment of donors, and lack of infrastructure. One typical example of limited blood transfusion capacity was noted in the study by Adetoro [42], in which it was described how the blood bank at their hospital is open only for 8 hours daily. At all other times, blood must be retrieved from the blood bank of a larger hospital, located 4 kilometers away. Not only was distance an issue, but the authors also found that the larger hospital’s blood bank frequently suffered a lack of blood supply, and husbands and other relatives were unwilling to donate blood when asked.

The main limitation of this systematic review derives from limitations of the individual audits. As mentioned above, the assessment of avoidable factors in many cases would have benefited from a greater degree of granularity. For instance, factors such as “substandard health worker practice” and “poor antenatal care” provide a broad orientation to where deficiencies in care occur, but greater specificity (e.g., what specific substandard heath worker practice took place? Why was antenatal care poor?) would provide more precise direction in order to successfully inform health solutions that target existing gaps in the health care system. Therefore, while the quantitative analyses presented are useful for helping to appreciate the relative frequencies with which avoidable factors were reported, the results of this study may have greater qualitative value through the description of where and when preventable deficiencies in care were reported to have occurred.

A second limitation was the lack of standardized audit forms used in the different studies included in this analysis. Only 25 studies used a standardized form within their own study and none of these were identical between studies. This likely resulted in differences regarding how factors were both identified and labeled.

A third limitation is that the analysis was limited to published datasets in the medical literature. As a result, the sample of deaths audited was not random. The majority of the datasets included were from Africa, and data were not weighted based on population size.

Finally, a limitation of this review is the number of datasets included. While the number of datasets identified was robust on an absolute scale, we were surprised that more did not exist given that the inclusion criteria were liberal in terms of study date (1965 onwards) and geography (any low- and lower-middle income country). Audits are a proven method for helping to improve quality of care relating to childbirth [68], and some countries, such as South Africa in particular, have taken pioneering efforts to incorporate audits into the national healthcare system [12, 13, 18]. The findings of this review that relatively so few audits are being conducted (or, at least, reported) globally could constitute a call to action for stakeholders to initiate mortality audit programs, particularly in settings where rates of maternal and perinatal deaths are high.

## Conclusions

Audit processes highlight precisely where breakdowns in clinical care occur and are an established method for facilitating quality improvement in health systems. Through a systematic review of avoidable factors in maternal and perinatal mortality we are able to identify the specific timing and nature of factors that are reported to be associated with severe childbirth-related harm in low resource settings. These data can be used to inform the development of health system improvements that specifically target known deficiencies in care, which represents a rational approach for measurably improving health and achieving progress towards Millennium Development Goals Four and Five.

## Electronic supplementary material

Additional file 1: **Preferred Reporting Items for Systematic Reviews and Meta-Analyses (PRISMA) Checklist.** (PDF 71 KB)

Additional file 2: **Estimate of contribution of substandard health worker practice to maternal and perinatal deaths.** (DOC 152 KB)

## Abbreviations

MDG: Millennium development goal
WHO: World Health Organization.
