# Supplementary material for: Audit-identified avoidable factors in maternal and perinatal deaths in low resource settings: a systematic review
Source: BMC Pregnancy Childbirth. 2014 Aug 16;14:280. doi: 10.1186/1471-2393-14-280 (PMC4143551; doi:10.1186/1471-2393-14-280)
Supplement: Supplementary file 2 — Additional file 2: Estimate of contribution of substandard health worker practice to maternal and perinatal deaths. (DOC 152 KB) [file 12884_2013_1154_MOESM2_ESM.doc]

**Estimate of contribution of substandard health worker practice to maternal and perinatal deaths**

| Author, Year | Estimate of contribution to deaths; % (CI) |  |
| --- | --- | --- |
| Ozumba, 2008 | 27.7 (15.6-42.6) | 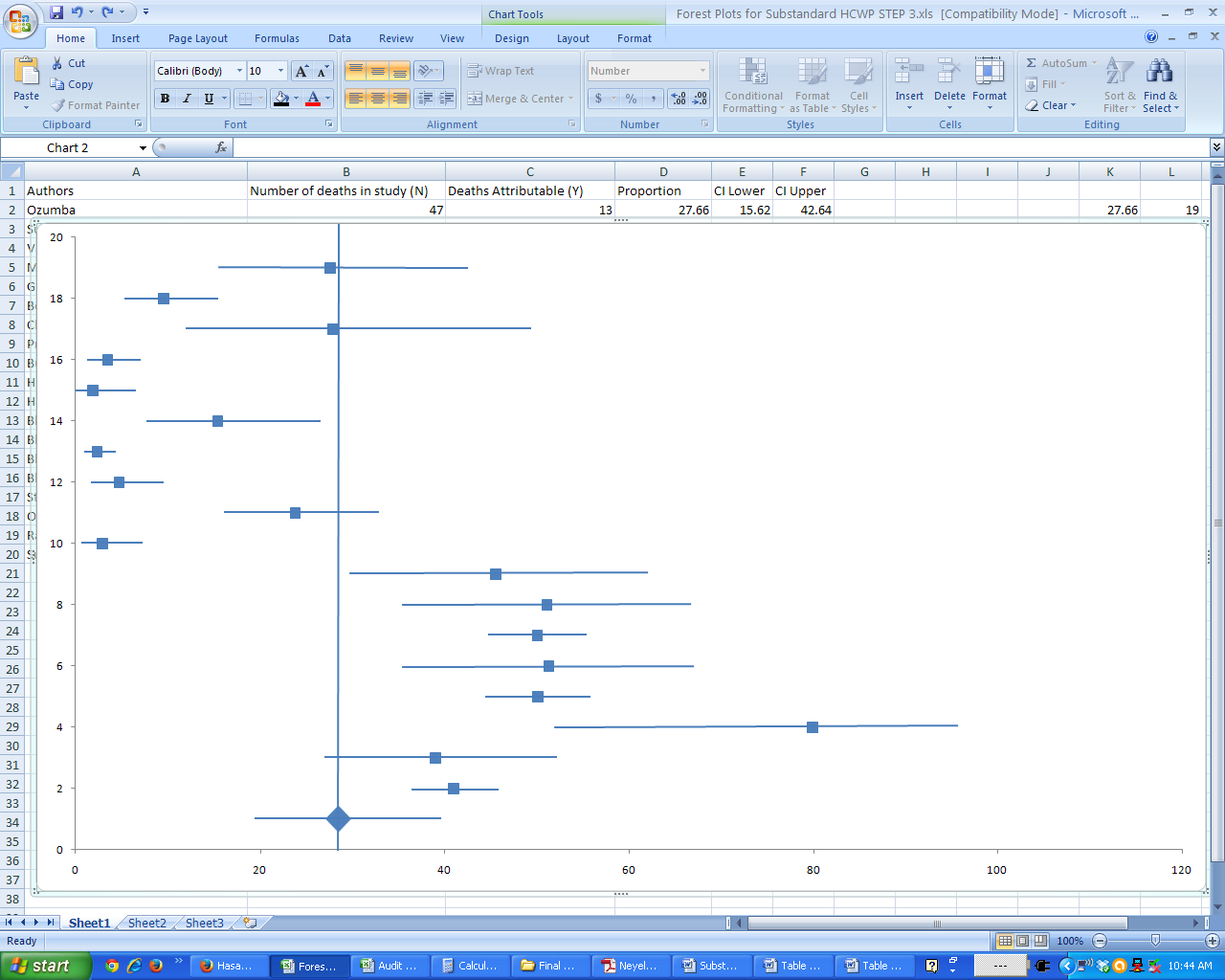 |
| Suprakito, 2002 | 9.6 (5.4-15.6) |
| Vangeenderhuysen, 1995 | 28.0 (12.1-49.4) |
| Mbarku, 2009 | 3.5 (1.4-7.1) |
| Granja, 2000 | 1.9 (0.2-6.7) |
| Bouvier-Colle, 2001 | 15.5 (7.8-26.7) |
| Chigbu, 2009 | 2.4 (1.1-4.5) |
| Price, 1984 | 4.8 (1.9-9.9) |
| Bullough, 1981 | 23.9 (16.2-33.0) |
| Hinderaker, 2003 | 2.9 (0.8-7.4) |
| Hailu, 2009 | 45.6 (29.8-62.1) |
| Bhatt, 1989^1^ | 51.2 (35.5-66.7) |
| Bhatt, 1989^2^ | 50.2 (44.9-55.4) |
| Bhatt, 1989^3^ | 51.4 (35.5-67.1) |
| Bhatt, 1989^4^ | 50.2 (44.5-55.8) |
| Steklenberg, 2002 | 80.0 (51.9-95.7) |
| Ouedraogo, 2002 | 39.1 (27.0-52.3) |
| Rachid, 2011 | 41.1 (36.4-45.8) |
| Effect Summary | 28.5 (19.5-39.7) |
|  |  |

^1^Maternal dataset from 1967-1968

^2^Perinatal dataset from 1967-1968

^3^Maternal dataset from 1983-1984

^4^Perintal dataset from 1983-1984
